# Infant VEPs reveal neural correlates of implicit naming: Lateralized differences between lexicalized versus name-unknown pictures

**DOI:** 10.1016/j.neuropsychologia.2015.07.027

**Published:** 2015-10

**Authors:** Suzy J. Styles, Kim Plunkett, Mihaela D. Duta

**Affiliations:** a Department of Experimental Psychology, University of Oxford, Tinbergen Building, 9 South Parks Road, OX1 3UD, UK; b Division of Psychology, Nanyang Technological University, 14 Nanyang Drive, 637332, Singapore

**Keywords:** Implicit naming, Event-related potentials, Lateralization, Infancy

## Abstract

Recent behavioural studies with toddlers have demonstrated that simply viewing a picture in silence triggers a cascade of linguistic processing which activates a representation of the picture’s name (Mani and Plunkett, 2010, 2011). Electrophysiological studies have also shown that viewing a picture modulates the auditory evoked potentials (AEPs) triggered by later speech, from early in the second year of life (Duta et al., 2012; Friedrich and Friederici, 2005; Mani et al., 2011) further supporting the notion that picture viewing gives rise to a representation of the picture’s name against which later speech can be matched. However, little is known about how and when the implicit name arises during picture viewing, or about the electrophysiological activity which supports this linguistic process.

We report differences in the visual evoked potentials (VEPs) of fourteen-month-old infants who saw photographs of animals and objects, some of which were name-known (lexicalized), while waiting for an auditory label to be presented. During silent picture viewing, lateralized neural activity was selectively triggered by lexicalized items, as compared to nameless items. Lexicalized items generated a short-lasting negative-going deflection over frontal, left centro-temporal, and left occipital regions shortly after the picture appeared (126–225 ms). A positive deflection was also observed over the right hemisphere (particularly centro-temporal regions) in a later, longer-lasting window (421–720 ms). The lateralization of these differences in the VEP suggests the possible involvement of linguistic processes during picture viewing, and may reflect activity involved in the implicit activation of the picture’s name.

## Highlights

• Infants’ visual evoked potentials (VEPs) to pictures differ when name is known.
• Lateralization suggests infant linguistic processing during silent picture viewing.
• Two stages identified in the neural correlates of implicit naming in 14-month-olds.

## Graphical abstract

**Figure:**
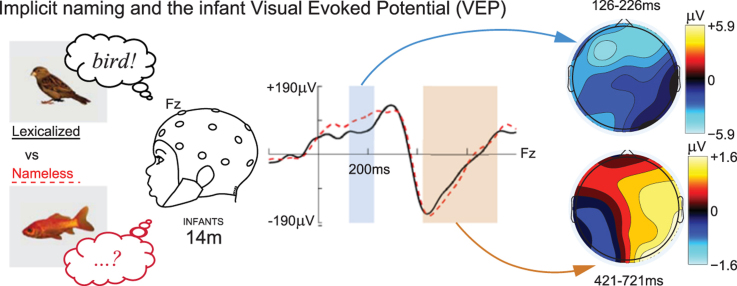

## 1 Introduction

For adults, viewing a picture sets in train a series of perceptual and cognitive processes that can trigger an automatic cascade of linguistic processing (Cutting and Ferriera, 1999; Morsella and Miozzo, 2002), including information about the phonology of a picture’s label (Schriefers et al., 1990). These phonological representations can arise even in the absence of direct fixation (Morgan and Meyer, 2005) or attention (Meyer and Damian, 2007; Morsella and Miozzo, 2002; Navarette and Costa, 2005), highlighting the automaticity of ‘implicit name’ generation. Phonological priming studies demonstrate that viewing a single picture triggers implicit naming even in early childhood (e.g., Mani and Plunkett, 2010), as the phonology of the implicit name influences down-stream auditory speech processing. For example, viewing a picture of a banana inhibits recognition of the word *bird*, but not *car* in 24-month-olds (Mani and Plunkett, 2011). These findings demonstrate that viewing a picture in silence induces linguistic processing of the picture’s label for young children, even when there is no requirement to produce the picture’s name. However, the process of implicit naming is not yet well understood, and the neural correlates of this process have yet to be documented.

Three areas of electrophysiological research provide evidence concerning linguistic processes which occur during passive picture viewing. Although none clarify the process of implicit naming itself, they provide valuable information about the likely time-course of language processing during picture viewing. A first line of research concerns the adult lateralized readiness potential (LRP), when decisions about whether to push a button (go/no-go), and which hand to use (L/R), must be made according to a picture’s semantics, and the phonology of its basic-level label. In a task which guided participants to prepare to name each of a series of pictures, but included occasional button-press trials, decisions based on semantics were complete by 150 ms, while decisions based on phoneme identity started to take their effect from 190 ms (van Turennout et al., 1997). Similar effects have also been observed in the absence of speech planning (Schmitt et al., 2000). These studies provide evidence that phonological information becomes available shortly after the onset of picture viewing – with phonological identity integrated into other cognitive processes within a few hundred milliseconds. As these studies rely on classifications arising from phoneme identity (e.g., consonant/vowel), they do not elucidate the neural processes of generating the implicit name. However, they suggest that internally generated phonological representations become classifiable within 200 ms of the onset of a picture presented in silence.

In a second branch of research, event-related potential (ERP) studies employing picture–speech mismatch paradigms have shown that auditory evoked potentials (AEPs) triggered by speech differ in their N400 amplitude according to how well incoming speech matches a currently viewed picture (Jescheniak et al., 2002), effects which have been replicated in infants (e.g., Friedrich and Friederici, 2004). One interpretation of these effects is that viewing a picture in silence allows the participant to generate an internal representation of the picture’s name, against which incoming speech is matched. These implicit names have been shown to be phonologically precise to the level of individual phonemes, in both adults and infants (Duta et al., 2012; Mani et al., 2011).

The detection of auditory mismatches appears to be comprised of two discrete stages, with early, short-lasting effects indexing violation of phonological expectations, discrete from a later, longer-lasting stage of semantic integration in adults (Desroches et al., 2008; Newman and Connolly, 2009; Steinhauer and Connolly, 2008), where the latter is consistent with the well known literature on adult N400 effects (for review, see Kutas and Federmeier (2011)). In one study of AEPs triggered by speech during picture viewing, responses to correct labels were compared to mismatches differing only in the height of a medial vowel, or to completely novel pseudowords (Duta et al., 2012). For those contrasts which differed from the onset of the first speech segment, adults and 14-month-olds showed short-lasting, early effects over the main peaks of the AEP, which were consistent with violation of phonological expectations (Adults: 80–180 ms; 180–280 ms; Infants: 150–250 ms), followed by a later, longer-lasting effect consistent with N400-type semantic integration (Adults: 370–670 ms; Infants: 400–600 ms). Although Duta et al. (2012) investigated the AEP in response to speech in a picture context, rather than the neurological substrates of implicit naming itself, the study demonstrated that phonological encoding of representations must be complete in the linguistic systems of 14-month-olds in under 1000 ms (the duration of the picture presentation prior to the labelling event) in order to support fast auditory mismatch detection (in this case 150–250 ms), providing further evidence that phonological representations become active during short periods of silent picture viewing, even in infancy.

A third branch of evidence concerning neural activity during picture viewing is the modulation of gamma-band activation, understood to be related to object processing. Gliga et al. (2010) investigated the gamma band activity of 12-month-old infants viewing pictures of items which were familiar or unfamiliar, and name-known or unknown. In their first study, three lists of items were selected, one list of familiar animals, food and objects whose names were likely to be *known* to the infants; a second list of familiar animals, food and household objects whose names were likely to be *unknown* to the infants, and a third list of insects, instruments, food and objects which were likely to be *unfamiliar* to the infants. For each participant, the infant’s parents selected items matching the experimental criterion from each list, and an equal number of each type were used as stimuli for test. During the test, pictures were presented in silence, and the authors report that the lexical status of the picture influenced gamma band activity (20–60 Hz) in a 500–800 ms window after picture onset: when the picture was nameable, gamma band activity increased over left posterior regions relative to a vertex reference. The authors interpret this lateralized gamma-band activity as modulation of object processing, caused by knowing the name of the object. To unpack whether the effect could have been driven by differential familiarity of the items in the different lists, they also replicated the effect of lexical status in a second experiment, with a set of novel objects whose exposure was experimentally controlled to include presentation *with labels* for one group of objects, presentation *without labels* for another, and no presentation (*completely novel*) for a third group of objects. In both experiments, no difference was observed between completely novel objects and familiar-but-nameless items, demonstrating that the observed differences in gamma-band activity were not due simply to differential visual familiarity.

Notably, the authors highlight that the timing of the observed effect corresponds to a period which has been shown to index semantic integration between words and pictures in N400-style picture-word mismatch studies for 12-month-olds (Friedrich and Friederici, 2005) and 14-month-olds (Duta et al., 2012; Mani et al., 2011), suggesting that the gamma-band modulation may have its origins in a semantic integration process. Gliga et al. (2010) also point out that the lateralization of the effect further suggests the involvement of linguistic systems – an interpretation that fits well with the changes in lateralization that are understood to emerge as the infant vocabulary grows (Mills et al., 2005) and as individual words are acquired by infants (Franklin et al., 2008).

However, two features of the Gliga et al. (2010) task limit the potential interpretation of the effect with regard to implicit naming. Firstly, since the presentation of all test pictures was silent, it is unclear whether the 12-month-olds in their study would have been guided to implicitly name the pictures. Secondly, given the temporal limitations of gamma-band oscillatory dynamics, it is unclear whether this approach would have been able to capture fine temporal details of a linguistic effect emerging over time, especially given the short-lasting nature of some phonological processing effects in infancy (i.e., Duta et al., 2012).

We report an ERP study which targets short-lasting effects which may emerge over the infant visual evoked potential (VEP), comparing responses to lexicalized versus nameless pictures, in a context known to induce implicit naming in infants: we present previously unpublished data collected during the visual presentation period of an infant picture-speech matching study for 14-month-olds (Duta et al., 2012). Pictures were presented on screen in silence for 1000 ms, after which a token of speech was presented. In this paper, we assess the lexical status of each item according to parental report of lexical comprehension – classifying each picture as lexicalized or nameless for each individual. Thus, each test item was lexicalized for some infants, but nameless for others, and could thereby act as its own visual control between participants.

While the original study was not designed for this purpose, the silent picture-viewing period provides a unique opportunity to investigate neural activity in response to pictures, in a context where infants are known to make predictions about upcoming speech, and the outcomes of implicit naming have been well documented. We predicted that the labelling context would guide toddlers to implicitly name each picture in the one second viewing time available, but this would only be possible in cases where children knew the picture’s name. We report both short-lasting and longer-lasting modulations of the VEP, which show lateralized differences in activity in cases where the picture is lexicalized, when compared to cases where the picture is nameless.

## 2 Materials and methods

### 2.1 Participants

We report results from fifteen 14-month-old infants from monolingual English speaking families (Median age: 14.2 months; Range: 13.8–14.5 months). Parents of participants were asked to fill in a vocabulary inventory (Hamilton et al., 2000) in the week preceding their experimental visit. The vocabulary inventory asked about which words from a fixed list the child ‘understands’. This inventory has previously been shown to have sensitivity at the level of individual items, for parents in the local community (Styles and Plunkett, 2009). A separate group of 24 parents (infant age range: 13.2–14.2 months) were asked to fill in an online survey judging the familiarity of the objects selected for test. Participants were recruited from a pool of families who had previously expressed interest in participating in developmental studies, and participants who attended an experimental visit were offered a gift for their participation. The study was approved by the Central University Research Ethics Committee.

### 2.2 Stimuli

Stimuli were sixteen nameable items which would be familiar to 14-month-olds, but whose names may or may not be known by participants. The familiarity of all items (with the exception of the body parts *hand* and *foot*) was normed using the Toddler Object Experience Survey (TOES). In this survey, parents were asked to mark how often their child would typically see each item, rated on a six point scale: ‘Every day’, ‘Most days’, ‘Once a week’, ‘Once a month’, ‘Rarely’, and ‘Never’. They were instructed that pictures, toys and cartoons of the items should be included along with real exemplars of the test items. The 16 test items were embedded in a survey containing 127 items, designed to mask the test items, and to guide parents to use the entire scale from ‘Every day’ (e.g., *bath*, *window*) to ‘Rarely’ or ‘Never’ (e.g., *popcorn*, *submarine*). Table 1 gives the results of the normative familiarity for each test item used in the current study. The TOES established that all stimuli would be predicted to be familiar to 14-month-olds in the local area, as they were encountered often ('most days', or 'every day') by the majority of infants (>65%).

For the experimental session, each stimulus item was depicted by a high-resolution colour photograph of a typical exemplar of the category. Photographs were digitally edited to remove backgrounds, adjust colour, and remove distracting features (e.g. clothing labels), and were presented on a 5% grey background at 1024×768 pixels. Audio tokens were produced in citation form by a female native speaker of British English with a standard Southern accent, recorded in a single session in a sound-attenuating booth using a solid state recorder sampling at 44.1 kHz, in 16 bit stereo. Audio stimuli were filtered to remove hiss and hum, and edited to remove head and tail clicks using Goldwave 5.23.

### 2.3 Experimental design

**Fig. 1:**
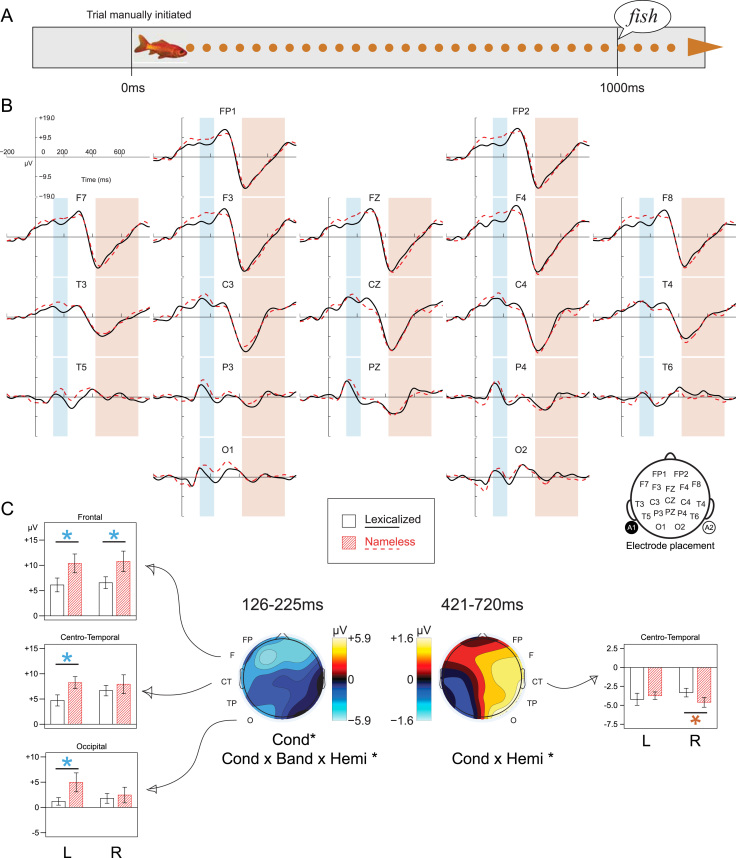
(A) Trial timeline. (B) Grand average ERP for lexicalized and nameless pictures at each sensor site, time-locked to onset of picture. Analysis windows overlayed (Early: 126–225 ms, Late: 421–720 ms). (C) Scalp localisation for effects in each time window: topographic maps of mean amplitude ERP difference (lexicalized minus nameless) in time windows of interest, with summary of main effects and interactions: *p<.05. Bar charts show mean ERPs averaged over time windows, for sensor regions where lexicalized and nameless stimuli elicited different responses, showing both hemispheres. *p<.05. Error Bars +/−1 S.E.

The electroencephalogram (EEG) was recorded while participants attended to a series of pictures of familiar animals and objects. Each trial consisted of the presentation of a picture on screen for 2500 ms. As shown in Fig. 1A, a labelling context was provided, which was expected to encourage implicit naming: after a fixed stimulus onset asynchrony (SOA) of 1000 ms, a token of speech was presented (for details, see Duta et al., 2012). The experimental presentation included at least six presentations of each picture, with repetitions separated by at least 14 other stimulus presentations. Unattended stimuli were marked during presentation, and were repeated at the end of the procedure if the infant was still attentive to the screen. The full stimulus set consisted of 96 trials. Different individuals were presented with between 71 and 160 trials.

### 2.4 Equipment

Visual stimuli were presented centrally on an LCD monitor 38×30 cm^2^, creating a viewing angle of approximately 26° from side to side. Audio stimuli were presented via two speakers centrally located above the screen. Stimuli were presented using Presentation (version 13.0.01.23.09, Neurobehavioral Systems Inc). Parallel port gamepads were used for stimulus triggering and online marking of unattended trials.

The EEG was recorded from 21 locations on the scalp using Compumedics Quik-Caps with Ag/AgCl sintered electrodes arranged according to the International 10–20 system of electrode placement (American Electroencephalographic Society, 1994). The VEOGL was measured with an electrode placed below the left eye, VEOGU was approximated by the signal recorded from FP1, while HEOGL and HEOGR were approximated using the signals recorded from F7 and F8. Quik-Cell sponges prepped with saline solution were used in all sensor sites, including facial and mastoid sites. The EEG and EOG signals were acquired with Neuroscan Scan 4.3 via NuAmps sampling at 1000 Hz. During recording, the signals were referenced to the left mastoid, and bandpass filtered for 0.1–100 Hz. At the start of each recording session, impedances of all electrodes were below 5 kΩ.

Artefact rejection and data processing were conducted offline in MATLAB (Version 7.7.0.471, R2008b, The MathWorks Inc., and Natick, MA) using EEGLab (Delorme and Makeig, 2004), and custom routines. Statistical analysis was performed in PASW Statistics (SPSS Inc, Version 18.0.3).

### 2.5 Procedure

Participants were tested in a specially designed EEG recording booth, where monitor and speakers were built into a plain grey wall, and no distractions were visible. After a short play-session in which the child was introduced to the EEG cap, and the cap and electrodes were positioned, infant participants sat facing the screen, in a caregiver’s lap. One experimenter monitored the signals from an adjacent control room, while a second experimenter monitored the behaviour of the infant from inside the EEG booth, in order to trigger each trial to start when the infant attended the screen, and to mark unattended trials for exclusion from analysis, using a hand-held gamepad. Testing sessions ended after presentation of the full stimulus set, or if infants became very active (e.g., talking, laughing, clapping), or otherwise ceased to attend to the screen.

To maintain infants’ attention to the screen, trials were manually interspersed with custom animations, the Soothers, Engagers and Eye-Catcher (SEE) cartoons (for details, see Duta et al., 2012).

### 2.6 Data processing

EEG signals were re-referenced to the average of the left and right mastoid channels. Custom zero-phase filters were applied (minimum-order Butterworth filters, high-pass: 3 dB attenuation at 1 Hz, 20 dB attenuation/octave; low-pass: 3 dB attenuation at 15 Hz, 40 dB attenuation at 50 Hz) to remove muscle and drift artefacts. The filtered EEG was segmented into VEP epochs containing a 200 ms baseline preceding visual onset, and 800 ms following visual onset (1000 ms in total). For each epoch, signals from each channel were baseline corrected to the median of the signal over the baseline period. Trials marked as unattended during data collection were excluded.

Finally, to detect and remove epochs corrupted by extreme values and ocular artefacts, custom Matlab routines automatically screened each epoch. Epochs were excluded if the signal from any electrode exceeded a threshold of 100 μV for maximum absolute actual value, or if the signal from the mastoid sites exceeded a threshold of 50 μV for maximum dynamic range. Ocular horizontal and vertical artefacts were detected by testing bipolar vertical EOG (VEOGL-VEOGU) and bipolar horizontal EOG (HEOGL-HEOGR) against a threshold of 75 μV. These exclusions resulted in a trial rejection rate of 60%. Following these exclusions, infants who gave less than 10 attended artefact-free trials per condition were removed from the original sample of 39 infants. This resulted in 15 participants providing an average number of 23 trials in the lexicalized condition (Range: 11–48) and 30 in the nameless condition (Range: 11–90). The total number of trials include per infant averaged 54 (Range: 27–105).^2^

### 2.7 Data analysis

We predicted that generating an implicit name for a lexicalized picture would trigger short-lasting differences in the infant VEP when compared to a nameless picture, and given the speed of known phonological processing effects in infants and adults (Duta et al., 2012; Schmitt, et al., 2000; van Turennout, et al., 1997), we expected these differences to emerge around 100–200 ms after the onset of the visual stimulus. Furthermore, we predicted lexicalized pictures would generate later, longer-lasting differences in the infant VEP, emerging between 400–500 ms, which may indicate modulation of object processing, as reported by Gliga et al. (2010), or other processes arising from generation of an implicit label.

Analyses were conducted on mean ERP amplitude values calculated over fixed time windows, in which VEPs to lexicalized pictures were compared to VEPs to nameless pictures. Statistical analysis was performed using Lexical Status×Band×Hemisphere repeated measures ANOVAs for which the scalp was divided into the five coronal bands, subdivided by hemisphere: frontal pole (FP1/FP2), frontal (F7, F3/F4, F8), centro-temporal (T3, C3/C4, T4), parieto-temporal (T5, P3/P4, T6), occipital (O1/O2) (see Fig. 1B for electrode placement, and Duta et al. (2012) for further details). Greenhouse–Geisser corrections have been applied where required (adjusted *F*-ratios are reported as *F*_G_).

Since this analysis constitutes an exploratory reanalysis, of a new paradigm, to identify the precise time windows for analysis for the predicted early and late effects, following Duta et al. (2012), we used an objective algorithm based on analysis of variance over consecutive bins to identify the precise onsets and offsets of the windows of interest to capture these effects. The algorithm consisted of a series of repeated measures ANOVAs comparing the influence of lexical status on mean amplitude ERP (subdivided by band and hemisphere, as above) over rolling bins of fixed duration, with onsets staggered every 5 ms.

Windows of interest were defined as those bins for which the *F*-value reached a local maximum, and effects or interactions with condition were significant below the *α*-level of .05. To capture short-lasting phonological effects predicted to occur early in picture processing, 100 ms bins were used. To capture longer-lasting effects predicted to occur around half way through the epoch, 300 ms windows were used. The rolling bins approach identified two windows of interest in which differences between experimental conditions were evident: 126–225 ms, and 421–720 ms.

## 3 Results

### 3.1 Item variables

Toddlers participating in the experimental visit were reported to understand a median of 99 words from the standardized vocabulary inventory (Range: 30–207), and to produce 8 (Range: 1–22). For the purpose of assessing the lexical status of each stimulus item for each infant participant, stimulus words marked in the CDI as ‘understood’ or ‘understood and also said’ were treated as ‘lexicalized’, while those which remained unmarked were treated as nameless. One stimulus, *ball,* was ‘lexicalized’ for all of the infants, and was removed from further analyses. Of the 15 remaining stimuli, participants knew the names of approximately half of the pictures used in the study (Median=9, Range: 2–12). Thus, for all individuals, some test items were lexicalized and others were nameless.

### 3.2 Visual evoked potentials

Fig. 1B presents the event-related potential (ERP) from each sensor site, with grand average ERPs to lexicalized and unknown stimuli shown separately. A sharp polarity inversion characteristic of an infant VEP is visible for both stimulus types at around 300–500 ms, maximally detected over frontal sites relative to the averaged mastoid reference. The difference VEP (lexicalized minus nameless) was computed for each individual, and Fig. 1C shows topographic maps of the average mean amplitude difference in each time window of interest.

#### 3.2.1 Early window: 126–225 ms

The VEP differed across the scalp, with posterior regions exhibiting a more pronounced negative-going local deflection than frontal regions during this window (Band: *F*_G_(1.40, 19.64)=13.59, *p*=.001, $\eta_{\mathrm{part}}^{2}$=.49). Lexicalized pictures elicited more negativity than pictures with no known label (Cond: *F*(1,14)=5.74, *p*=.03, $\eta_{\mathrm{part}}^{2}$=.29), and this effect differed across the scalp (Cond x Band x Hemi: *F*(4, 56)=3.18, *p*=.02, $\eta_{\mathrm{part}}^{2}$=.19), with lexicalized pictures eliciting significantly more negativity than nameless pictures over frontal regions, left centro-temporal, and left occipital regions (F7/F3/F4/F8: *t*(14)=2.32, *p*=.03, *d*=.7; T3/C3: *t*(14)=2.52, *p*=.03, *d*=.8; O1: *t*(14)=2.41, *p*=.03, *d*= 0.7).

#### 3.2.2 Late window: 421–721 ms

The VEP differed across the scalp, with frontal regions exhibiting more negativity than posterior regions relative to an average mastoid reference during this window (Band: *F*_G_(1.34, 18.76)=25.88, *p*<.001, $\eta_{\mathrm{part}}^{2}$=.65). Responses to pictures in the two conditions differed between the hemispheres (Cond×Hemi: *F*(1.14)=6.49, *p*=.02, $\eta_{\mathrm{part}}^{2}$=.32), with lexicalized pictures eliciting more positivity than unknown pictures on the right, as compared to slightly less on the left, although neither of these comparisons reached the alpha-level of .05 when tested independently (Right: *t*(14)=1.69, n.s., *d*=.5; Left: *t*(14)=.45, n.s., *d*=.2). Another way of understanding this interaction is that the hemispheric difference had a different polarity for nameless as compared to lexicalized pictures. The difference between lexicalized and nameless pictures was observed most strongly over right centro-temporal regions (C4/T4: *t*(14)=2.37, *p*=.03, *d*=.6).

## 4 Discussion

These findings demonstrate that lexical status modulates infants' processing of pictures presented in a silent, pre-labelling context, with lateralized effects differentiating lexicalized from nameless items. This modulation of ERP occurs in a context previously demonstrated to generate expectation-related differences in AEPs following picture labelling. In the previous study (Duta et al., 2012), 14-month-olds were able to detect fine phonological mismatches to a picture’s expected name, suggesting that picture viewing allowed a detailed phonological representation of the picture’s name to be generated – an ‘implicit name’ – against which incoming speech was then matched. By exploring differences between the lexicalized and the nameless pictures during the silent pre-naming period, this investigative analysis of the picture viewing time allows new insights into neural activity which supports the implicit naming process, thereby facilitating phonological mismatch detection.

The early difference between lexicalized and nameless pictures (126–225 ms) was recorded more strongly in frontal and left centro-temporal/occipital regions. The left-lateralization of the early effect is consistent with reports from the infancy literature, that linguistic processing may shift to the left hemisphere as words are learned (Mills et al., 2005), an effect which is evident even in the absence of ostensive labelling (Franklin et al., 2008). The timing of this effect is consistent with known phonological processes in adults, which are known to arise within the first 200 ms of picture viewing (Schmitt et al., 2000; van Turennout et al., 1997).

In the later, longer time window (421–720 ms), differences between lexicalized and nameless items were also observed, with a clear hemispheric lateralization, recorded most strongly over right centro-temporal regions. The separation in time from the first effect, and the differences in scalp distribution suggest that the two effects reflect discrete stages in processing of pictures. This late window begins slightly ahead of the Gamma-band oscillatory effects reported by Gliga et al. (2010), suggesting that the effects reported here may represent a level of processing which precedes and contributes to the modulation of gamma-band oscillations.

While the timing of this second effect is similar to N400-like effects in toddlers’ responses to auditory speech in a picture context, (Duta et al., 2012; Friedrich and Friederici, 2005; Mani et al., 2011), the scalp distribution is quite different, suggesting this later effect has a different origin. As the hemispheric difference had opposite polarity for lexicalized and nameless pictures, this second effect may have been driven by linguistic processes surrounding *not knowing* the label – the effects in this time window may represent activity related to preparing to learn, update or check possible names against the upcoming word-form. Since the current paradigm involved the presentation of auditory labels in all trials, every presentation of a picture whose name is not known constitutes a possible learning opportunity.

The fact that the late difference between lexicalized and nameless words was observed most strongly in sites over the right hemisphere is consistent with models of hemispheric lateralization as an emergent property of dynamic language learning. In Minagawa-Kawai et al. (2011) review of evidence for competing models of hemispheric lateralization of language, they review a long history of evidence that structural characteristics of the auditory cortex in the left hemisphere are best suited to the spectro-temporal detail required to discriminate fine-grained phonetic contrasts, while the right-hemisphere is better suited to larger scale temporal units of speech, including prosody and melody (c.f., also Johnsrude et al., 1997; McGettigan and Scott, 2012; Schwartz and Tallal, 1980; Shankweiler and Studdert-Kennedy, 1967; Zatorre et al., 2002). The review also notes that the hemispheres differ in their bias for processing of different types of information, with both humans and non-human animals showing a left-hemisphere advantage for abstract categorical processing, and a right-hemisphere advantage for exemplar-based processing – even for non-linguistic stimuli (c.f. also Curby et al., 2004; Marsolek and Burgund, 2008; Yamazaki et al., 2007). Minagawa-Kawai et al. go on to argue that the interaction between biological properties of the auditory cortex, along with the bias for abstract representation, combine over individual development to produce the familiar left-hemispheric bias for linguistic processing observed in the majority of adults.

According to this model, as ‘exemplar based’ linguistic information is accumulated, processing of some types of linguistic information would be expected to shift from the right hemisphere to the left hemisphere, with increasing abstraction of linguistic categories. Evidence from AEPs for infants at different ages has shown developmental changes in lateralization of word-level processing: Following the onset of a word, 13–17 month old infants show early (200–350 ms) bilateral differences in their AEPs for known versus unknown words, followed by a later difference (600–800 ms), observed only in the right hemisphere, while older children (20-month-olds), show only the early, left hemisphere effects (Mills et al., 2005). Furthermore, when experience with individual words is controlled (Mills et al., 2005), 20-month-old infants with smaller vocabularies show bilateral activation differences (200–500 ms) between completely novel words, and the same words following training. Infants the same age with larger vocabularies showed a larger post-training response difference in the left hemisphere – suggesting an interaction between word-learning expertise and the lateralization of individual word-form representations. That is to say, experienced word learners increasingly shift the processing of an individual word-form from the (exemplar-based) right hemisphere to the (abstract) left hemisphere with only a short period of familiarisation.

While the infants in the current study are younger than the 20-month-olds reported in the novel word exposure study by Mills and colleagues, and the studies themselves differ in whether they report auditory evoked potentials in response to speech stimuli, or visual evoked potentials for lexicalized versus nameless items, there is good reason to suppose that the late (421–720 ms) right hemisphere activation differences we observe in the current study may be related to this shift in information processing: The late effect could reflect preparatory activity related to the expectation of an upcoming word-form (a learning opportunity), or it could represent a partial reactivation of prior speech encounters – exemplars which have not yet been fully encoded as a lexical category.

It is indeed possible that some learning was taking place during the study itself. However, learning was unlikely to be successful in this context, since the original study was designed to investigate phonological mismatches, and the verbal labels were only correct approximately one third of the time (for details see Duta et al., 2012). Furthermore, even if some learning did take place over the course of the study, for some infants, on some words, it was insufficient to overshadow the measurable differences in the ERPs overall. Future studies featuring systematic presentation of auditory labels could be used to investigate how the VEP prior to word onset changes over the course of an experimental study as learning progresses.

In the current context, it was not possible to ascertain directly whether the modulation we observe in the VEP predicts the later modulation of the AEP in response to naming, because the population included in the current study is different from the previously reported group, due to attentional and behavioural factors (e.g., where one infant may have blinked often following the onset of pictures, another may have blinked often following the onset of speech), as well as linguistic factors (e.g., infants with artefact-free trials in both the timing of the AEP and the VEP may have been excluded from the current investigation because they knew too many, or too few of the test words). Rather, this investigation must be treated as an exploratory re-analysis: An investigation into the kinds of activation patterns which occur while viewing lexicalized versus nameless pictures, in a context where implicit naming is known to occur, albeit for different infants. However, the analysis provides targets in terms of timing and scalp distribution for future investigations into the neural correlates of implicit naming in infants.

The current study used a norming study to evaluate that the items selected for use in the test would be likely to be familiar to the majority of infants, based on data from the local community. It is therefore possible that some of the differences in the signals arise out of individual item familiarity rather than lexicalization per se. However, the study by Gliga et al. (2010) demonstrated that lateralized object processing in silence was not altered by item familiarity, but only by whether or not the item had a known name, making it likely that lexicalization was the main driver of the effects we observe here.

Taken together, these results suggest that there are discrete stages in the infant response to lexicalized versus nameless items, and these effects may be related to the implicit naming process known to occur in this context. Since silent picture viewing has not previously been investigated for the neural correlates of lexicalization in ERPs, there are no established time windows in which experimental effects would be reliably predicted. For this reason, we implemented an objective algorithm to identify windows of interest as driven by the structure of the current data set. This statistical approach is less common than more familiar methods, but has the advantage of being unbiased by theoretical expectations (i.e., ‘windows’ may appear at times outside the expected ranges). Algorithms of this kind are typically uncorrected for multiple comparisons, and since this is the first time these windows have been reported, the results should be treated as novel targets for future investigations until they have been replicated.

Three features of the current study may have contributed to the observation of these effects. First, the use of a time-sensitive method to investigate the detailed temporal structure of the VEP during passive picture viewing allowed us to observe two stages in the 14-month-old response to lexicalized pictures. Secondly, the inclusion of a labelling context in the current study may have guided infants to form a phonological representation against which upcoming speech could then be matched. Thirdly, by focussing on comprehension vocabulary, we found that despite the infants’ small productive vocabularies (producing less than 22 words), the lexical status of items in their comprehension vocabulary (as reported by their parents) triggered a difference in the VEPs consistent with the generation of an implicit name for the picture.

## Conflicts of interest

The authors declare no conflicts of interest.

## Figures and Tables

**Table 1:** Stimulus list.

Item	% 14-month-olds who see this item most days/every day^a^	% 14-month-olds who see this item at least once a week^a^	% Participants who understand item’s name^b^
Bib	75	75	60
Bird	83	92	67
Block	92	100	20
Bottle	100	100	53
Bread	100	100	40
Cat	79	92	87
Dog	79	96	93
Doll	67	88	27
Fish	67	96	53
Fridge	96	100	20
Foot^c^	–	–	53
Hat	67	92	33
Hand^c^	–	–	40
Sock	100	100	60
Spoon	100	100	53

a Percentage of 14-month-olds in the local area, according to the TOES survey.

b Percentage of participants in the current study for whom the item was reported as ‘understood’ or ‘understood and also said,’ according to the Oxford CDI completed in the week before test.

c Body-parts ‘hand’ and ‘foot’ were not included in the TOES survey.

## Appendix

See Appendix Table 1.

## Footnotes

2 Artefact rejection routines for the current investigation were run over the timing of the VEP, rather than the AEP, as in Duta et al. (2012). This resulted in a different sample of trials from a group of participants which was partially overlapping with, but substantially different from, the previously reported study.
